# Ovarian tissue cryopreservation followed by controlled ovarian stimulation and pick-up of mature oocytes does not impair the number or quality of retrieved oocytes

**DOI:** 10.1186/s13048-014-0080-8

**Published:** 2014-08-26

**Authors:** Marie-Madeleine Dolmans, Maria-Laura Marotta, Céline Pirard, Jacques Donnez, Olivier Donnez

**Affiliations:** Université Catholique de Louvain, Institut de Recherche Expérimentale et Clinique, and Cliniques Universitaires Saint-Luc, Gynecology Department, 1200 Brussels, Belgium; Université Catholique de Louvain, Institut de Recherche Expérimentale et Clinique, and CHU Mont-Godinne, Gynecology Department, 5530 Yvoir, Belgium; Société de Recherche pour l’Infertilité (SRI), 1150 Brussels, Belgium

**Keywords:** Fertility preservation, Ovarian cortex cryopreservation, Embryo cryopreservation

## Abstract

**Background:**

The objective of this study was to evaluate the feasibility of fertility preservation in cancer patients by combined bilateral ovarian cortex cryopreservation and embryo freezing.

**Methods:**

This was a cohort-controlled study in a university hospital center. Sixteen patients with a recent cancer diagnosis were included in the study. They all consented to fertility preservation by a combined technique: ovarian tissue cryopreservation (OTC) followed by ovarian stimulation for in vitro fertilization (IVF) and embryo freezing. The control group included 100 women of the same age undergoing IVF for male factor infertility.

**Results:**

The mean number of metaphase II oocytes was 8.3 per patient (±7.7) and was not statistically different from the control group (8.1 ± 5.6). The mean number of good quality embryos obtained was not statistically different in the 2 groups (4.2 versus 4.4).

**Conclusion:**

OTC before embryo freezing does not impair the number or quality of cryopreserved embryos, but increases fertility preservation potential.

## Introduction

In recent decades, improvements in cancer survival rates in children and young adults have stimulated interest in fertility preservation (FP) techniques. Indeed, therapies which greatly increase life expectancy also accelerate oocyte depletion, leading to early menopause [1]. This risk of premature ovarian failure depends on the age of the patient at the time of gonadotoxic treatment and the type of chemo- and/or radiotherapy protocol (dose and number of cycles) applied [1–3]. Several options are currently available to preserve fertility in patients with cancer, including embryo cryopreservation, oocyte cryopreservation and ovarian tissue cryopreservation (OTC). The American Society for Reproductive Medicine (ASRM) has endorsed embryo and oocyte cryopreservation, but still considers OTC an experimental technique [4–7], even if in some conditions, especially in prepubertal girls or when immediate chemotherapy is required, there is no alternative at present [7–9].

There are, however, some limitations to performing oocyte or embryo cryopreservation in cancer patients. Studies by Rienzi et al. and Cobo et al. show that around 20 oocytes are required to achieve a live birth [4,5]. This number can be obtained in egg donation programs or in case of FP for social reasons, but rarely in women with cancer. Even when a delay in treatment is possible, it is usually for no more than one cycle [5,6]. Thus, the good results obtained in egg donation programs cannot be extrapolated to cancer patients, nor can the quality of eggs be guaranteed in these women [5,7].

To maximize the chances of FP, we propose a combined technique: 1) OTC and 2) in vitro fertilization (IVF) treatment to cryopreserve embryos.

The aim of this study was to evaluate if bilateral biopsy and OTC followed by controlled ovarian stimulation (COS) is a feasible method to preserve fertility without any negative impact on the number of oocytes.

## Patients and methods

### Patients

After adequate counseling, patients referred to our institution for FP were offered cryopreservation of both ovarian cortex and embryos, depending on their marital status, the type of cancer, and the time interval before beginning oncological treatment [7].

Sixteen patients (study group) suffering from cancer and undergoing OTC followed by COS were included in the study (Table 1). All the women had regular ovulatory cycles (25–32 days) at the time of diagnosis. Eight patients were diagnosed with hematological malignancies (Hodgkin’s disease (n = 6), non-Hodgkin’s disease (n = 2)), and the rest with infiltrating ductal carcinoma (n = 4), colon cancer (n = 2), ovarian cancer (n = 1) and abdominal Ewing’s sarcoma (n = 1). They all needed chemotherapy (+ radiotherapy in 7 cases) (Table 1). It is important to note that none of them had undergone chemotherapy or radiotherapy before FP.

**Table 1:** **Characteristics of cancer patients, type of cancer and adjuvant treament**

	**Age**	**Type of cancer**	**Type of chemotherapy**	**Body or pelvic irradiation**
1	26	Ovarian carcinoma	Carbotaxol	-
2	25	NHD(a)	ABVD	-
3	26	HD(b)	ABVD	30 Gy
4	23	HD(b)	ABVD	-
5	24	HD(b)	R-CHOP	-
6	28	Colon cancer	FOLFOX	-
7	31	IDC(c)	Unknown	Breast
8	25	Abdominal Ewing’s sarcoma	VIDE	45 Gy
9	32	NHD(a)	CHOPP	-
10	25	HD(b)	BEACOPP + ABVD	-
11	34	IDC(c)	Unknown	Breast
12	23	HD(b)	BEACOPP + ABVD	-
13	34	IDC(c)	FEC	Breast
14	21	HD(b)	Unknown	Unknown
15	32	IDC(c)	FEC – taxotere	Breast
16	24	Colon cancer	FOLFOX	-

(a)NHD: non-Hodgkin’s disease.

(b)HD: Hodgkin’s disease.

(c)IDC: infiltrating ductal carcinoma.

The control group (n = 100) included age-matched patients undergoing IVF treatment for male factor infertility. Intracytoplasmic sperm injection (ICSI) was required for all control group cases (inclusion criteria: Kruger morphology <5%).

Patients referred for FP in our institution were seen in an emergency context. The OTC protocol was approved by the institutional review board of the Cliniques universitaires Saint-Luc. After obtaining informed consent, bilateral biopsy by laparoscopy and OTC were performed as soon as possible. COS was started one or two days before laparoscopy or on the same day. The type of stimulation administered depended on the patient cycle phase at the time of laparoscopy. Patients in the follicular phase received a short agonist or antagonist protocol, while those in the luteal phase received a long agonist protocol. Gonadotropins used were recombinant (Gonal-F®, Merck-Serono, Darmstadt, Germany) or urinary purified (Menopur®, Ferring, Kiel, Germany). Trigger was performed by subcutaneous injection of human chorionic gonadotropin (hCG) (Pregnyl®, MSD, Hertforshire, UK) when the dominant follicle reached at least 15-16 mm in size. Based on their cycle phase, 3 study patients and 9 control patients were assigned a long agonist protocol, 3 study patients and 4 control patients a short agonist protocol, and 10 study patients and 87 control patients an antagonist protocol. The 4 patients suffering from breast cancer were older than the others (range: 31-34 y) and did not receive any specific stimulation treatment such as letrozole.

### Consent

Patients gave their informed consent for their medical and administrative data to be communicated to external bodies in a coded manner.

### Statistical analyses

Data are presented as mean +/−standard deviation. Statistical analyses were performed using the Mann–Whitney U-test with GraphPad Prism (version 4.00 for Windows, GraphPad Software, San Diego, California, USA, www.graphpad.com). A *p*-value of less than 0.05 was considered statistically significant.

## Results

Patient age was 27.1 ± 4.2 years (range: 21-34 y) in the study group and 28 ± 2.6 years (range: 21-33 y) in the control group, which was not statistically different (p = 0.1744) (Table 2).

**Table 2:** **Patient and stimulation characteristics and outcome**

	**Study group (n = 16)**	**Control group (n = 100)**	**p value**
**Age (years)**	27.1 y (+/− 4.2)	28 (+/− 2.6)	0.1744 ns
**Stimulation duration**	10.1 days (+/− 2.8)	10.8 days (+/− 3.3)	0.7687 ns
**Total dose of gonadotropins**	2440 IU (+/− 994)	1681 IU (+/− 695)	0.0017*
**E2 level on the D**_**hCG**_	1350 pg/ml (+/− 1742)	959 pg/ml (+/− 713)	0.8596 ns
**Number of retrieved oocytes**	10.7 (+/− 9.9)	10.8 (+/− 6.2)	0.3716 ns
**Number of mature oocytes (MII)**	8.3 (+/−7.7)	8.1 (+/− 5.6)	0.5812 ns
**Number of good quality embryos**	4.2 (+/− 5.0)	4.4 (+/− 4.1)	0.1676 ns

Data are presented as mean +/- standard deviation. D_hCG_: day of human chorionic gonadotropin injection/E2: estradiol/IVF: in vitro fertilization/MII: metaphase II/ns: not statistically significant.

(*)statistically significant (*p*-value < 0.05).

The duration of stimulation was 10.1 ± 2.8 days in the study group and 10.8 ± 3.3 days in the control group, with no statistically significant difference (n = 0.7687) (Table 2). The total dose of gonadotropins used was 2440 ± 994 IU and 1681 ± 695 IU in the study and control groups respectively. The total dose of gonadotropins used was statistically higher in the study group (*p* = 0.0017*). The estradiol level reached at the time of hCG trigger was 1350 ± 1742 pg/ml in the study group and 959 ± 713 pg/ml in the control group, showing no statistically significant difference between the 2 groups (p = 0.8596) (Table 2).

### Oocyte pick-up

The number of collected oocytes was not statistically different between the 2 groups, with 10.7 ± 9.9 oocytes per study patient and 10.8 ± 6.2 oocytes per control patient (p = 0.3716) (Table 2). The number of mature oocytes (MII) per patient was 8.3 ± 7.7 in the study group and 8.1 ± 5.6 in the control group, again showing no statistically significant difference (p = 0.5812).

### Fertilization results

In the study group, all mature oocytes were microinjected with the partner’s spermatozoa by ICSI to avoid the risk of failed fertilization. In the control group, all couples underwent ICSI for male factor infertility.

In the study group, the number of good quality embryos obtained per patient was 4.2 ± 5.0 and they were all cryopreserved on day 2 or 3. In the control group, 4.4 ± 4.1 good quality embryos were obtained per patient on day 3 (Table 2). There was no statistically significant difference between the 2 groups (p = 0.1676).

### Breast cancer patients

The ovarian response was variable among the 4 patients suffering from breast cancer, so no statistical analyses could be carried out. One patient had no embryos cryopreserved, while another had 17 embryos cryopreserved.

## Discussion

Cryopreservation of embryos is the most widely established method of FP, but is essentially applicable to adult women with a stable male partner [7,10–14]. In the literature, survival rates of cryopreserved embryos and vitrified mature oocytes after thawing are reported to be 80-90%, while pregnancy rates are comparable to those recorded with fresh oocytes [4,5].

However, it should be pointed out that these results were obtained in non-cancer patients and that adequate numbers of oocytes need to be collected. Recent reviews by Rienzi et al. [4] and Cobo et al. [5] suggest that around 20 mature oocytes are required to achieve a live birth. An immediate obstacle is the fact that only one COS cycle can usually be performed in cancer patients, yielding a ‘relatively’ low number of oocytes (and embryos). For this reason, results from egg donation programs cannot be extrapolated to cancer patients, nor can the quality of oocytes be guaranteed.

Very little literature is available on pregnancy rates in cancer survivors after frozen-thawed embryo transfer, because the majority of embryos frozen for FP have not yet been transferred [5,10,13–16]. Barcroft et al. recently published their 15-year follow-up of patients undergoing embryo cryopreservation for FP, obtaining 2 live births and 1 miscarriage after 9 embryo transfers in 5 patients [16].

In women with cancer, embryo or oocyte cryopreservation techniques yield a limited number of embryos for transfer [6,17], and there is often only time to perform one COS cycle before starting oncological treatment [6,7,10,18]. Combining these techniques with OTC could therefore be considered an alternative approach.

In our study, 8.3 and 8.1 MII oocytes were respectively retrieved from cancer patients and control patients (of the same mean age), and the number of good quality embryos obtained was respectively 4.2 ± 5 and 4.4 ± 4.1. Our study proves that OTC performed just before the start of COS or even on day 1 or 2 of stimulation does not affect the number of mature oocytes and embryos obtained, which was similar to the control group.

In the literature, two studies by Huober-Zeeb et al. [19] and Dittrich et al. [20] report the results of a combined technique (OTC and COS) for FP, but using two different sequences.

Huober-Zeeb et al. [19] compared two cancer patient groups. In the study group, they cryopreserved half of one ovary, followed by COS and IVF, and compared their results with the control group, who also underwent COS and IVF treatment for FP, but without previous OTC. They did not find any difference between these 2 cancer groups in terms of duration of stimulation (mean 10.2 days vs 10.6 days in the study and control groups respectively), total dose of gonadotropins, or total number of retrieved oocytes per patient (mean 12.1 vs 13.1).

Very recently, Dittrich et al. [20] conducted a retrospective study in cancer patients with cryopreservation of half of one ovary on the same day as oocyte pick-up (OPU) after IVF stimulation. The average number of oocytes retrieved per patient was 10, and 67% of them were successfully fertilized by ICSI. The authors did not note any perioperative complications, but it is known that vascularization of the corpus luteum is greater in the early luteal phase. It should be pointed out that OPU 34-36 hours after hCG administration followed by ovarian tissue biopsy may cause severe bleeding due to the presence of multiple earlier corpora lutea, which are very highly vascularized. It was indeed reported that, for the same reason, the ovarian tissue was of poor quality for cryopreservation (C.Y. Andersen, personal communication).

Ovarian stimulation requires time and it is recognized that this procedure needs to be performed before the first cycle of chemotherapy [21]. Dolmans and colleagues clearly demonstrated that only very few poor quality embryos were obtained when IVF was attempted immediately after one or two courses of chemotherapy. Ovarian stimulation should therefore no longer be offered for OPU after chemotherapy. Moreover, Meirow et al. reported high rates of malformation in offspring after treatment with cyclophosphamide (in an experimental model) [22]. In our study, the time between OTC and OPU was short (mean 14.8 days). This interval could be further shortened using modified IVF treatments, such as antagonist injection during the luteal phase to induce luteolysis [6,10,23–28].

Removal of approximately 50% of one ovary for cryopreservation in Dittrich’s study had only a limited impact on the number, quality and fertilization potential of oocytes. When compared with our study, which investigated the number of MII oocytes and embryos in cancer patients, we could add that removal of approximately 20% of ovarian cortex from each ovary did not result in fewer oocytes the control group, leading us to suggest that it is maybe more appropriate to remove no more than 20% if IVF and embryo cryopreservation are subsequently planned. No bleeding was observed after OPU in our study, nor in that of Huober-Zeeb et al. [19].

Concerning the number of oocytes retrieved from cancer patients, there is still some debate. A meta-analysis concluded that the mean number of oocytes is lower in women with cancer [29], but Tulandi and Holzer found that malignancies do not affect the number of oocytes [30]. Our study, although limited, failed to observe any significant difference between cancer and non-cancer patients of the same age. Nevertheless, due to this discrepancy, it is strongly advised that patients be informed that the number of oocytes may be lower than expected due to the accompanying neoplasm [6]. The total dose of gonadotropins used in the study group was significantly higher than in the control group. Reasons for this include the potential risk of obtaining fewer embryos because of the neoplasm, concerns about having no embryos to cryopreserve in patients able to undergo only one cycle, and the desire to cryopreserve as many embryos as possible. However, none of our patients had to delay their oncological treatment due to hyperstimulation syndrome. Indeed, using GnRH agonists to trigger ovulation before OPU has been shown to dramatically lower the risk of ovarian hyperstimulation syndrome [6,10,24] and shorten the luteal phase, possibly allowing a second COS if feasible [18,28,31,32].

Theoretically, IVF treatments induce high estradiol levels that could be deleterious to hormone-sensitive cancers like breast cancer. To avoid this effect, ovarian stimulation with letrozole and antagonist has been proposed [31]. In this case, the letrozole or antagonist should be continued after OPU to precipitate a decrease in estradiol levels [10,31,32]. In our series, even in non-metastatic breast cancer and lymph node-negative breast cancer, classical COS was used, as we (gynecologists and oncologists) consider that there is no evidence that 4–5 days of high estradiol levels may be deleterious for patients or impact their survival.

## Conclusion

In the present study, we demonstrate that cryopreservation of bilateral ovarian cortex followed by IVF treatment is a feasible and safe approach to preserve fertility before oncological treatment. The number of cryopreserved embryos obtained was not statistically different from the control group and not affected by the previous bilateral biopsy for OTC. Moreover, this technique does not delay oncological treatment. It may even be proposed to patients without a male partner in association with oocyte vitrification.
